# Specialized Hydrocarbonoclastic Bacteria Prevailing in Seawater around a Port in the Strait of Malacca

**DOI:** 10.1371/journal.pone.0066594

**Published:** 2013-06-18

**Authors:** Maki Teramoto, Shu Yeong Queck, Kouhei Ohnishi

**Affiliations:** 1 Oceanography Section, Kochi University, Kohasu, Oko, Nankoku, Kochi, Japan; 2 School of Chemical & Life Sciences, Nanyang Polytechnic, Singapore, Singapore; 3 Research Institute of Molecular Genetics, Kochi University, Nankoku, Kochi, Japan; Universidad de Salamanca, Spain

## Abstract

Major degraders of petroleum hydrocarbons in tropical seas have been indicated only by laboratory culturing and never through observing the bacterial community structure in actual environments. To demonstrate the major degraders of petroleum hydrocarbons spilt in actual tropical seas, indigenous bacterial community in seawater at Sentosa (close to a port) and East Coast Park (far from a port) in Singapore was analyzed. Bacterial species was more diverse at Sentosa than at the Park, and the composition was different: *γ*-*Proteobacteria* (57.3%) dominated at Sentosa, while they did not at the Park. Specialized hydrocarbonoclastic bacteria (SHCB), which use limited carbon sources with a preference for petroleum hydrocarbons, were found as abundant species at Sentosa, indicating petroleum contamination. On the other hand, SHCB were not the abundant species at the Park. The abundant species of SHCB at Sentosa were *Oleibacter marinus* and *Alcanivorax* species (strain 2A75 type), which have previously been indicated by laboratory culturing as important petroleum-aliphatic-hydrocarbon degraders in tropical seas. Together with the fact that SHCB have been identified as major degraders of petroleum hydrocarbons in marine environments, these results demonstrate that the *O. marinus* and *Alcanivorax* species (strain 2A75 type) would be major degraders of petroleum aliphatic hydrocarbons spilt in actual tropical seas.

## Introduction

The Strait of Malacca is a major route for crude oil shipments, and the port of Singapore is the busiest hub for transhipment in the world. In accord with this, the port of Singapore has been reported to be chronically contaminated by petroleum oil [1]. In addition, some part of the Strait has shallow water depth, which is prone to stranding of ships and crude oil pollution. Petroleum hydrocarbons accidentally discharged into seawater are degraded mainly by bacteria, and these petroleum-hydrocarbon-degrading bacteria appear to be ubiquitous in marine environments.

A wide variety of the petroleum-hydrocarbon-degrading bacteria are known to date [2], [3], and the degradation mechanisms are rather well understood [4]. Most hydrocarbonoclastic bacteria metabolize either aliphatic or aromatic hydrocarbons, although some bacteria such as strains of *Pseudomonas* [5] and *Rhodococcus* [6] have been shown to degrade both types of hydrocarbons. Among hydrocarbonoclastic bacteria, *Alcanivorax* [7]–[11] and *Cycloclasticus* [12]–[14] have been identified as key microorganisms with major roles in the degradation of aliphatic and aromatic hydrocarbons, respectively, in marine environments [15]. *Alcanivorax* and *Cycloclasticus* use limited carbon sources with a preference for petroleum hydrocarbons and thus are specialized hydrocarbonoclastic bacteria (SHCB; [15]). Accordingly, abundance of the SHCB can be an indicator for the petroleum hydrocarbon contamination in their surrounding environment. *Alcanivorax* strains are distributed in natural marine environments around the world [16]. On the other hand, *Cycloclasticus* strains have been suggested to preferentially inhabit colder seawater [17]–[19].

The petroleum-hydrocarbon-degrading bacteria characterized are mainly from temperate marine environments and relatively few studies have been conducted on the bacteria in tropical marine environments. However, recently *Oleibacter* strains, which are also SHCB, have been indicated as major degraders of aliphatic hydrocarbons spilt in tropical marine environments [20], [21]. In addition to *Oleibacter* bacteria, *Alcanivorax* species (such as strain 2A75 type) have been indicated to be also important for aliphatic-hydrocarbon degradation in tropical marine environments [20]. On the other hand, another *Alcanivorax* species, *Alcanivorax borkumensis*, can be considered as the major degrader in temperate marine environments [8], [11], [22]. The important, major degraders of petroleum hydrocarbons in tropical marine environments have been indicated only by laboratory culturing and never through observing the bacterial community structure in actual tropical marine environments. The result derived from an environment that simulated a natural environment could be different from the result derived from the actual natural environment. As SHCB have been identified to be major degraders of petroleum hydrocarbons in marine environments [15], we investigated the type of SHCB that are abundant in seawater around the port of Singapore to demonstrate the major degraders of petroleum hydrocarbons in actual tropical marine environments in the present study.

## Materials and Methods

### Ethics Statement

Permission was obtained for the described field study from National Environment Agency of Singapore government. The location was not privately owned or protected, and this field study did not involve endangered or protected species.

### Seawater Sample

Surface seawater was sampled at Sentosa beach (100 ml; 1°15′ N, 103°49′ E) and at East Coast Park (200 ml; 1°18′ N, 103°54′ E) in Singapore in December 2011. The seawater was filtered on a 0.22-µm pore size membrane (express plus, Millipore) to collect the bacteria in the seawater, and the membranes were completely dried at 60°C. The sampling, filtering and drying were done on the same day. The membranes were brought to Japan under dryness condition, and stock at –80°C before using them for DNA extraction.

### Construction of 16S rRNA Gene Fragment Library and Sequencing

DNA was extracted from the microorganisms on the membrane by ISOIL for Beads Beating (Nippon Gene, Tokyo) using a beads beater at 30 frequency/sec for 90 seconds (Mixer Mill MM 300; Retsch, Germany). PCR amplification of the 16S rRNA bacterial gene was performed using forward primer 27F (AGRGTTTGATCMTGGCTCAG; [23]) and reverse primer 519R (GWATTACCGCGGCKGCTG; [24]). The forward primer included the fusion primer A (CGTATCGCCTCCCTCGCGCCA) in its 5′ end, followed by a key sequence (TCAG) and sample specific 10 bp barcodes according to GS Junior Titanium emPCR Lib-A kit (Roche). Similarly, the reverse primer included the fusion primer B (CTATGCGCCTTGCCAGCCCGC) in its 5′ end, followed by the key sequence and sample specific 10 bp barcodes. The PCR was performed in a total volume of 25 µl containing 1.25 U *Taq* DNA polymerase (Ex Taq; Takara, Japan), Mg^2+^-containing buffer supplied with the polymerase (2 mM Mg^2+^ at the final concentration), 1 M betaine (Sigma), 1.25 µl DMSO (Sigma), 200 µM of each deoxynucleoside triphosphate, 1 µM of each primer, 5 ng of the extracted DNA. The amplification programme was as follows: (i) 5 min at 94°C; (ii) 30 cycles consisting of 1 min at 94°C, 1 min at 50°C and 2 min at 72°C; and (iv) a final extension for 7 min at 72°C. Four independent PCR amplifications were performed for each sample. Gel based analysis showed that PCR products of the expected size were amplified in equal concentration in each reaction. Equal volumes of the four reaction solutions for the same sample were mixed, and PCR products were purified using AMPure XP (Agencourt). Emulsion-based clonal amplification using Lib-A kit (Roche) and sequencing on the GS Junior 454 system (Roche) were performed according to the manufacturer’s instructions.

### Analysis of the Library

Tag and primer regions used for PCR were removed from the sequences by using QIIME (http://www.qiime.org/; [25]) through OTUMAMi (http://www.mwm.k.u-tokyo.ac.jp:8080/Plone?set_language=en) and manually with a sequence editing software Se-Al (http://tree.bio.ed.ac.uk/software/seal/) after alignment of sequences using ClustalX (version 2.1; [26]). The sequences were then checked for putative chimera sequences using Bellerophon server [27] and Mallard program [28] after aligned with the ClustalX. Sequences indicated to be chimera by the both programs were removed, and then the sequences were used for the bacterial community composition analysis in this study. The sequences used were deposited in DDBJ Sequence Read Archive (DRA) under accession numbers DRZ000062 and DRZ000063.

Ribosomal Database Project (RDP; [29]) was used for classifying the sequences into phylum/class, clustering the sequences into operational taxonomic units (OTUs), selecting representative sequences from each OTU, and calculating Chao 1 richness estimates [30], [31]. The representative sequences were compared to DNA sequences in the GenBank database by using BLAST [32].

### Strains

The reference strain *Alteromonas macleodii* NBRC 102226^T^, *Alcanivorax* sp. 2A75 ( = NBRC 105763; [20]) and *Altererythrobacter* sp. 1*l*E25 ( = NBRC 105748; [18]) were obtained from NBRC (NITE Biological Resource Center), while *Erythrobacter aquimaris* JCM 12189^T^ was from RIKEN BRC through the National Bio-Resource Project of the MEXT, Japan.

### Degradation of Crude Oil and GC-MS Analysis

The SW medium is comprised of (per litre) 1 l seawater, 1 g NH_4_NO_3_, 0.2 g K_2_HPO_4_ and 12 mg FeCl_3_ [20], while dMB plate is of (per litre) 0.9 l seawater, 0.1 l distilled water, 3.74 g Marine broth 2216 (Difco), and 15 g agar. Seawater for formulating media was collected from the surface at a coastal area of Muroto city, Kochi, Japan (33°18′ N, 134°11′ E).

The crude-oil-degrading activity was measured as described previously [20], unless stated otherwise. Cells of each bacterium (test strains, *A. macleodii* NBRC 102226^T^ and *E. aquimaris* JCM 12189^T^; positive control strains, the aliphatic-hydrocarbon degrader *Alcanivorax* sp. 2A75 [20] and an aromatic-hydrocarbon degrader *Altererythrobacter* sp. 1*l*E25 [18]) freshly grown on a dMB plate supplemented with 0.5% (w/v) pyruvate at 20°C were collected and suspended in filter-sterilized seawater to an OD_600_ of 0.5. Then 150 µl of this bacterial suspension was inoculated into 3 ml sterilized SW medium supplemented with 3 µl crude oil (heat-treated Arabian light crude oil; [20]). Large amounts of cells of the test two strains grown on the dMB plate with 0.5% (w/v) pyruvate in the same way were also inoculated into 3 ml sterilized ONR7a medium [12] supplemented with 3 µl crude oil. These cultures were incubated for 14–16 days with shaking (200 r.p.m.) at 30°C to examine the crude-oil-degrading abilities. Non-inoculated tubes were similarly incubated and served as non-degrading controls. Hydrocarbons were extracted with n-heptane. After dehydrated and concentrated, the extracts were subjected to GC-MS using a 6890N gas chromatograph with a 5973 mass-selective detector (Agilent Technologies). To investigate whether a strain degraded petroleum hydrocarbons, all peak areas for hydrocarbons obtained with the GC-MS selected ion monitoring were normalized by dividing by the peak area for 17α(*H*),21β(*H*)-hopane [33]. Aliphatic- and aromatic-hydrocarbon degrading activities were detected with the positive control strains 2A75 and 1*l*E25, respectively.

## Results and Discussion

### Diversity of Bacterial Species in Seawater at Sentosa and East Coast Park

**Figure 1:**
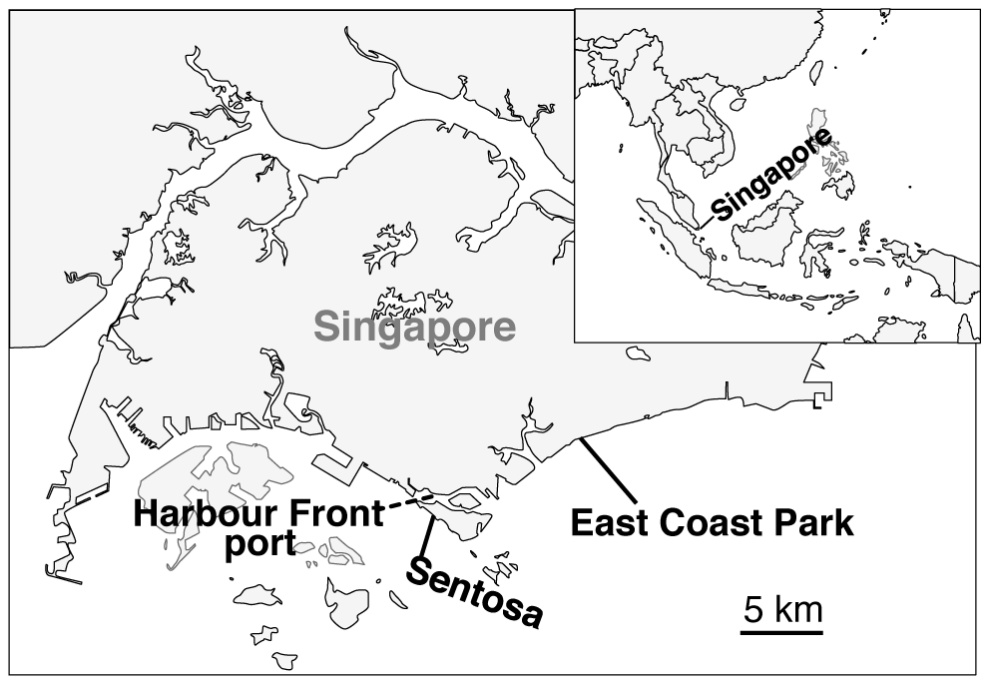
Two sampling sites, Sentosa and East Coast Park, for the seawater in Singapore.

**Figure 2:**
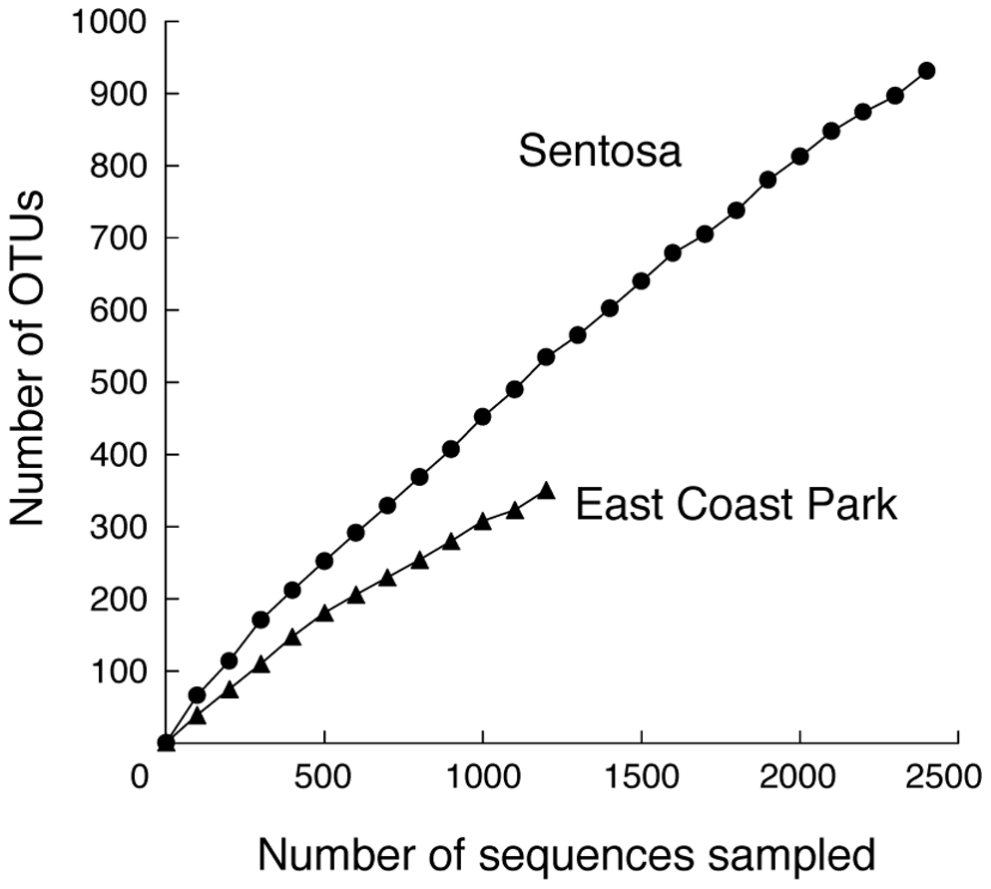
Rarefaction curves for bacteria in seawater at Sentosa and East Coast Park. The number of OTUs (maximum distance of 0.03) detected vs the number of sequences arbitrarily sampled is plotted.

**Figure 3:**
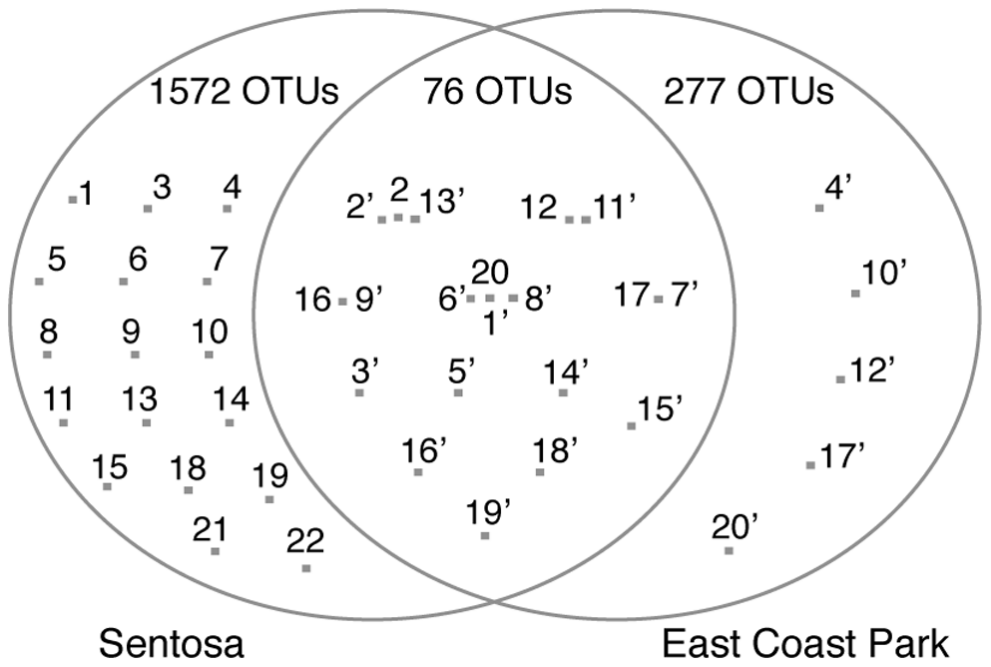
Venn diagram showing the OTU distributions between the seawater samples at Sentosa and East Coast Park. The distributions were estimated by the total and each numbers of OTUs from the two seawater samples (Table 1). Relations (identical, close or independent) of abundant OTUs from the two seawater samples (listed in Table 2 and 3) are also shown.

Seawater was sampled at Sentosa, which is close to Harbourfront port, and at East Coast Park (Fig. 1). The bacteria in seawater at Sentosa and East Coast Park were examined by sequencing the 16S rRNA gene fragments obtained from the seawater. The Chao1 OTU (operational taxonomic unit) richness estimates indicated that bacterial species in seawater at Sentosa were about three times as diverse as those at East Coast Park (Table 1). This increased diversity at Sentosa compared to East Coast Park was confirmed by their rarefaction curves (Fig. 2). In addition, out of 1925 OTUs from 6823 total sequences from both sites (Table 1), only a fraction of OTUs (76 OTUs) were shared by both sites (Fig. 3). Many bacterial species found only at Sentosa could include diverse petroleum-hydrocarbon-degrading bacteria and various bacteria that are tolerant to the crude oil and its concomitant heavy metals which would be general and main contaminants at ports [34].

**Table 1:** Number of sequences analyzed and OTUs (maximum distance of 0.03; species level grouping), and the Chao1 OTU richness estimates for the 16S rRNA gene fragments (404–524 bp) obtained from seawater at Sentosa (close to Harbourfront port) and at East Coast Park.

Sample	Sequences	OTUs	Chao 1
Sentosa	5606	1648	3855
East Coast Park	1217	353	1161
Total	6823	1925	

### Comparison of Bacteria in Seawater at the Two Sites at Phylum/class Level

**Figure 4:**
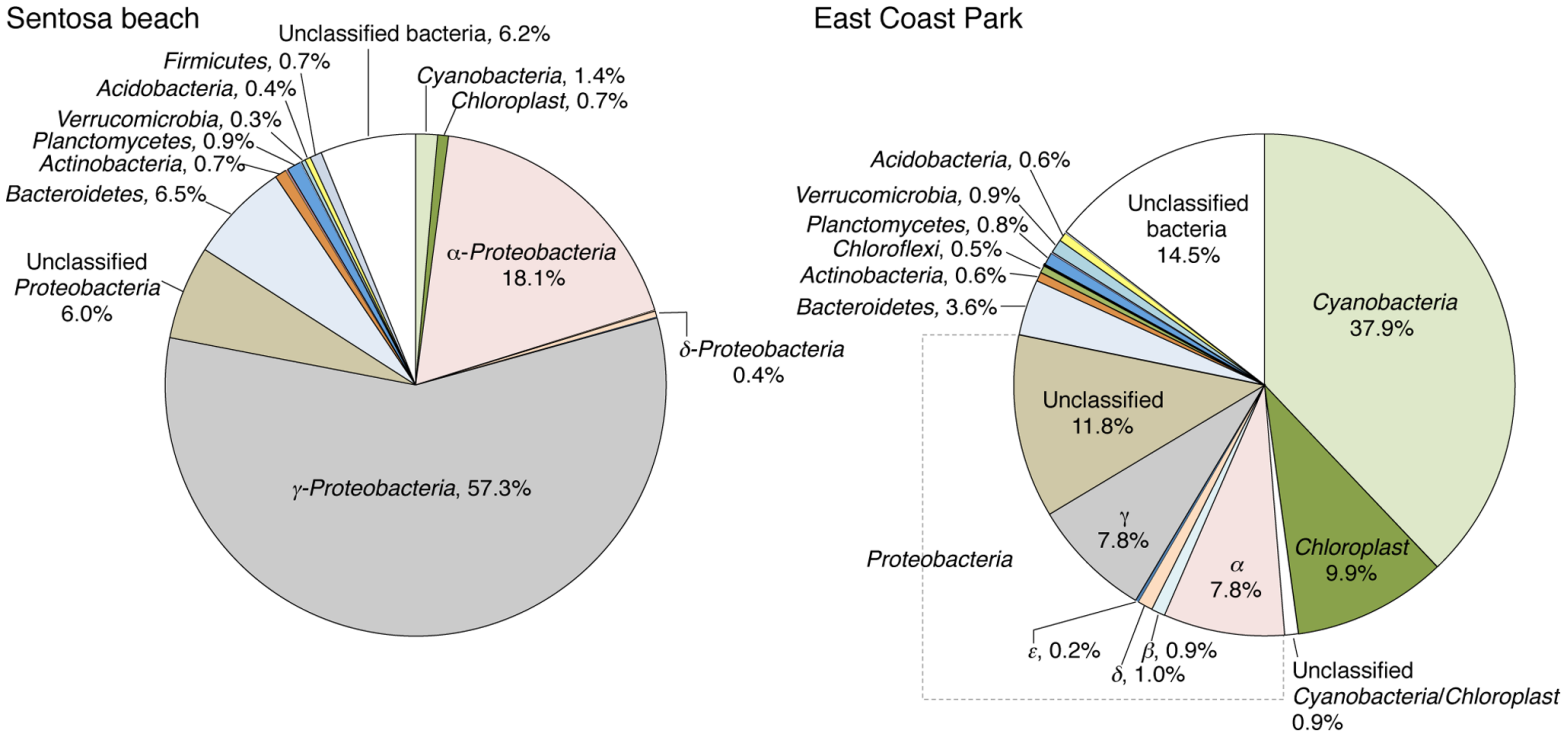
Bacterial community composition at phylum/class level in seawater at Sentosa and East Coast Park. Sequences were assigned to phylum or class with RDP classifier with a confidence threshold of 80%. Indications are given for the composition with a frequency of 0.1% or more.

The obtained sequences were assigned to phylum or class using RDP classifier with a confidence threshold of 80% (Fig. 4). At Sentosa, *γ*-*Proteobacteria* were the most dominant class, followed by *α*-*Proteobacteria* (Fig. 4). In most marine environments, *γ*-*Proteobacteria* appear outcompeted by *α*-*Proteobacteria* [35]–[37]. It has been shown that *γ*-*Proteobacteria* prefer elevated concentrations of nutrients and are outcompeted by *α*-*Proteobacteria* at low nutrient concentrations [38]. Our results thus suggest that the seawater around Sentosa would contain an elevated level of nutrients. On the other hand, bacterial community structure at East Coast Park was different from that at Sentosa, and *Cyanobacteria* which are unlikely to degrade petroleum hydrocarbons [39] were the most dominant group in seawater (Fig. 4).

### Abundant Bacterial Species and Petroleum-hydrocarbon-degrading Activity

SHCB, *Oleibacter marinus* and *Alcanivorax* species (strain 2A75 type), were found as abundant species at Sentosa (Table 2), indicating that seawater at Sentosa was likely to be contaminated with petroleum hydrocarbons. SHCB were not found as abundant species at East Coast Park (Table 3), and bacteria of *Alcanivorax* and *Oleibacter* were not detected at this site, suggesting that the level of crude oil would have been low in seawater at East Coast Park.

**Table 2:** Phylotypes of the abundant OTUs from the 16S rRNA gene fragment sequences from seawater at Sentosa constituting more than 0.5% of the total sequences.

OTU	Sequences	Closest GenBank relative to a representative of each OTU		Identity (%)
		Phylum/class	Strain, species, or clone (accession no.)	
1	625 (11.1%)	*γ-Proteo.*	*Alteromonas macleodii* ^T^ (AB681740)	98.3
2	170 (3.0%)	*α-Proteo.*	Uncul mar bac clone SW14 (JQ347423)	100
3	126 (2.2%)	*α*-*Proteo.*	*Erythrobacter aquimaris* ^T^ (NR_025789)	100
4	119 (2.1%)	*γ-Proteo.*	Lake bac K2-7 (AY345405)	99.4
5	103 (1.8%)	*γ-Proteo.*	*Marinobacter* sp. EM492 (GU223379)	98.6
6	102 (1.8%)	*α*-*Proteo.*	Uncul mar bac clone V-S-67 (JN018738)	99.5
7	102 (1.8%)	*γ-Proteo.*	Uncul *Alteromonas* sp. clone K4S103 (JN177672)	99.6
8	69 (1.2%)	*α*-*Proteo.*	*Oceanobacterium insulare* NBRC 102018 (AB681665)	100
9	67 (1.2%)	*γ-Proteo.*	*Pseudidiomarina* sp. MOLA 425 (AM990692)	98.2
10	62 (1.1%)	*γ-Proteo.*	*Marinomonas communis* ^T^ (AB681738)	99.8
11	62 (1.1%)	*Flavo.*	Uncul mar bac clone SHFG744 (FJ203324)	93.1
12	54 (1.0%)	–	Uncul mar bac clone IMS2D43 (JN233326)	100
13	54 (1.0%)	*γ-Proteo.*	*Vibrio natriegens* ^T^ (AB680922)	99.4
14	47 (0.8%)	*α*-*Proteo.*	*Sphingomonas* sp. NBRC 101717 (AB681541)	97.0
15	45 (0.8%)	*γ-Proteo.*	Uncul mar bac clone Pm_planulaeLM_E04 (FJ497177)	94.9
16	45 (0.8%)	*α*-*Proteo.*	Uncul mar bac clone DMS16SrDNA9 (JQ013151)	99.8
17	40 (0.7%)	*α*-*Proteo.*	Uncul mar bac clone SW6 (JQ347415)	100
18	36 (0.6%)	*γ-Proteo.*	*Oleibacter marinus* 1O18 (AB435651)	100
19	34 (0.6%)	*γ-Proteo.*	*Alcanivorax* sp. 2A75 (AB435642)	99.4
20	32 (0.6%)	*Cyano.*	*Synechococcus* sp. RS9916 (AY172826)	100
21	29 (0.5%)	*α*-*Proteo.*	Uncul mar bac clone SS_WC_13 (FJ973583)	99.5
22	29 (0.5%)	*γ-Proteo.*	Uncul *Aestuariibacter* sp. clone HAHS13.75 (HQ397070)	97.1

The 36.6% of the total 5606 sequences comprised the 22 most abundant OTUs in Table 1.

Identity values are based on 418–524 sequenced base pairs.

*Proteo.*, *Proteobacteria*; *Flavo.*, *Flavobacteria*; *Cyano.*, *Cyanobacteria*; –, unknown; Uncul, Uncultured; mar, marine; bac, bacterium; ^T^, type strain of the species.

**Table 3:** Phylotypes of the abundant OTUs from the 16S rRNA gene fragment sequences from seawater at East Coast Park constituting more than 0.5% of the total sequences.

OTU	Sequences	Closest GenBank relative to a representative of each OTU		Identity (%)
		Phylum/class	Strain, species, or clone (accession no.)	
1′	233 (19.1%)	*Cyano.*	*Synechococcus* sp. RS9916 (AY172826)	100
2′	99 (8.1%)	*Proteo*.	Uncul mar bac clone PEX_6-P_C3 (JF509023)	100
3′	78 (6.4%)	*Cyano.*	Uncul mar bac clone BAC-C111 (JN113083)	100
4′	53 (4.4%)	*Chl.*	Uncul mar bac clone OS2SD79 (JN233433)	100
5′	47 (3.9%)	*Cyano.*	*Cyanobium* sp. Suigetsu-CR5 (AB610889)	100
6′	34 (2.8%)	*Cyano.*	Uncul mar bac clone Reef_M07 (GU119187)	99.5
7′	31 (2.5%)	*α*-*Proteo.*	Uncul mar bac clone SW6 (JQ347415)	100
8′	28 (2.3%)	*Cyano.*	Uncul mar bac clone MWLSB53 (FJ937852)	100
9′	26 (2.1%)	*α*-*Proteo.*	Uncul mar bac clone DMS16SrDNA9 (JQ013151)	100
10′	19 (1.6%)	–	*Micromonas* sp. RCC299 mitochondrion (FJ859351)	84.7
11′	18 (1.5%)	–	Uncul mar bac clone DMS16SrDNA51 (JQ013164)	99.6
12′	14 (1.2%)	*Chl.*	Uncul mar bac clone SW-Oct-82 (HQ203792)	99.8
13′	14 (1.2%)	*α*-*Proteo.*	Uncul mar bac clone ARTE9_118 (GU230244)	100
14′	12 (1.0%)	*Flavo.*	Uncul mar bac clone N505B_36 (GU941050)	99.8
15′	11 (0.9%)	*Proteo.*	Uncul mar bac clone 6C233256 (EU805256)	99.5
16′	9 (0.7%)	*α*-*Proteo.*	Uncul mar bac clone OS2SD36 (JN233317)	99.8
17′	8 (0.7%)	*Chl.*	Uncul mar bac clone OO.P3.LT.32.ab1 (HQ821714)	100
18′	8 (0.7%)	*Chl.*	Uncul mar bac clone en1385-52 (JQ240691)	100
19′	7 (0.6%)	*γ-Proteo.*	Uncul mar bac clone XME27 (EF061959)	99.2
20′	7 (0.6%)	*Chl.*	Uncul mar bac clone N201B_349 (GU940745)	99.1

The 62.1% of the total 1217 sequences comprised the 20 most abundant OTUs in Table 1.

Identity values are based on 404–509 sequenced base pairs.

*Proteo.*, *Proteobacteria*; *Flavo.*, *Flavobacteria*; *Cyano.*, *Cyanobacteria*; *Chl.*, *Chloroplast*; –, unknown; Uncul, Uncultured; mar, marine; bac, bacterium.

Abundant species of genera *Marinobacter* [40], *Pseudidiomarina* [41], *Marinomonas* [42], *Vibrio* [43] and *Sphingomonas* [15] detected at Sentosa (Table 2) are potential petroleum-hydrocarbon degraders. These bacteria, with the possible exception of *Pseudidiomarina* (however, there is no report on the hydrocarbon-degrading activity from all the type strains of *Pseudidiomarina*), grow on a broad range of carbon sources [15], [44], [45]. Maybe the most and third abundant species of genera *Alteromonas* and *Erythrobacter*, respectively, detected at Sentosa (Table 2) would not degrade petroleum hydrocarbons, because no hydrocarbon-degrading activity was detected with type strains of *Alteromonas macleodii* and *Erythrobacter aquimaris* in this study. However, *Alteromonas* species have been found as main species in petroleum-contaminated marine environments [8], [46], and this reason is unknown. Therefore, the *O. marinus* and *Alcanivorax* species (strain 2A75 type) were strongly suggested to be the only SHCB detected as abundant species (constituting more than 0.5% of the total bacterial population) at Sentosa (Table 2), demonstrating that these species would be the major degraders of petroleum aliphatic hydrocarbons spilt in actual tropical marine environments. *A. borkumensis* was not detected at Sentosa, demonstrating that *A. borkumensis* might not become important *Alcanivorax* species for the degradation in actual tropical marine environments.

The most abundant species detected at East Coast Park, *Synechococcus* species (Table 3), may favor tropical marine environments, as dominance of *Synechococcus* has been observed in tropical seawater [35]. Abundant species found only at East Coast Park mostly belonged to class *Chloroplast* (Table 3, Fig. 3), species of which may not have been tolerant to contaminants around the port.

N and P are the primary growth limiting nutrients in marine environments. *A. macleodii* has grown on a N free medium [47], suggesting the possibility of its N_2_ fixation. This would be beneficial for its dominance (Table 2). Consistently, *A. macleodii* has become dominant in (Mediterranean offshore) seawater supplemented with glucose and P [38]. *A. macleodii* produces exopolysaccharides [48] that bind heavy metals [49], [50], which would be useful for the heavy metal remediation around the port.

Although the key aromatic-hydrocarbon degrader *Cycloclasticus* appears to prefer colder seawater [17]–[19], it has been detected from tropical seawater [18]. *Cycloclasticus* has constituted 0.6% of the total bacterial population in seawater at Tokyo Bay [14]. However, *Cycloclasticus* was not detected in this study, suggesting that its level was very low at two sites.

### Conclusions

It was demonstrated that *O. marinus* and *Alcanivorax* species (strain 2A75 type) but not *A. borkumensis* would be the major degraders of petroleum aliphatic hydrocarbons spilt in actual tropical marine environments. The *Alcanivorax* species (strain 2A75 type) would represent a novel species of *Alcanivorax* [20]. Description of this important species as the novel species will promote rational and systematic petroleum bioremediation strategies for tropical marine environments.
